# Seroprevalence of Cytomegalovirus, Epstein Barr Virus and Varicella Zoster Virus among Pregnant Women in Bradford: A Cohort Study

**DOI:** 10.1371/journal.pone.0081881

**Published:** 2013-11-27

**Authors:** Lucy Pembrey, Pauline Raynor, Paul Griffiths, Shelley Chaytor, John Wright, Andrew J. Hall

**Affiliations:** 1 Department of Infectious Disease Epidemiology, Faculty of Epidemiology and Population Health, London School of Hygiene and Tropical Medicine, London, United Kingdom; 2 Bradford Institute for Health Research, Bradford, United Kingdom; 3 Centre for Virology, University College London Medical School, London, United Kingdom; 4 Virology Department, Royal Free Hospital, London, United Kingdom; University of Regensburg, Germany

## Abstract

**Objective:**

To estimate the seroprevalence of cytomegalovirus (CMV), Epstein Barr virus (EBV) and varicella zoster virus (VZV) among pregnant women in Bradford by ethnic group and country of birth.

**Methods:**

A stratified random sample of 949 pregnant women enrolled in the Born in Bradford birth cohort was selected to ensure sufficient numbers of White UK born women, Asian UK born women and Asian women born in Asia. Serum samples taken at 24-28 weeks’ gestation were tested for CMV IgG, EBV IgG and VZV IgG. Each woman completed a questionnaire which included socio-demographic information.

**Results:**

CMV seroprevalence was 49% among the White British women, 89% among South Asian UK born women and 98% among South Asian women born in South Asia. These differences remained after adjusting for socio-demographic factors. In contrast, VZV seroprevalence was 95% among women born in the UK but significantly lower at 90% among South Asian women born in Asia. EBV seroprevalence was 94% overall and did not vary by ethnic group/country of birth.

**Conclusions:**

Although about half of White British women are at risk of primary CMV infection in pregnancy and the associated increased risk of congenital infection, most congenital CMV infections are likely to be in children born to South Asian women with non-primary infection during pregnancy. South Asian women born in South Asia are at risk of VZV infection during pregnancy which could produce congenital varicella syndrome or perinatal chickenpox. Differences in CMV and VZV seroprevalence by ethnic group and country of birth must be taken into account when universal immunisation against these viruses is contemplated.

## Introduction

Cytomegalovirus (CMV), Epstein Barr virus (EBV) and varicella zoster virus (VZV) are common herpesviruses which cause persistent infections usually acquired during childhood. Cytomegalovirus is the most common congenital infection in the UK affecting around 3 per 1000 births [1] and can cause neurological impairment such as hearing loss [2]. In utero transmission of CMV can occur following primary maternal infection during pregnancy but can also occur in women with natural immunity, either because they reactivate latent virus or become reinfected with a different strain [3]. CMV can also be transmitted perinatally from mother to child where it is usually asymptomatic, except in premature babies [4]. Postnatally, CMV is transmitted from mother to child through breastfeeding and close contact. In developed countries like the UK where breastfeeding is less prevalent and of shorter duration than in developing countries, child to child transmission of CMV is common in day care and similar settings [5]. Early childhood infection with CMV is usually asymptomatic or causes only mild, flu-like symptoms. Uninfected adults in regular contact with young children are at risk of CMV infection, of particular significance for female childcare workers of childbearing age. 

EBV is rarely transmitted in utero. EBV infection is usually asymptomatic or causes mild, flu-like symptoms in early childhood but can cause glandular fever if acquired in adolescence or adulthood [6]. In utero transmission of VZV is also rare but can cause congenital varicella syndrome or perinatal chickenpox [7]. In childhood VZV causes chickenpox, but children may present with herpes zoster (shingles) without preceding chickenpox if their first exposure to this virus was in utero [8]. 

VZV vaccine is available but not part of the routine childhood vaccination programme in the UK although has recently been introduced for adults in their 70s to prevent shingles. Vaccines are being developed for CMV [9,10] and EBV [11,12]. To inform potential vaccination programmes, it is essential to understand the current epidemiology of these infections in childhood. Maternal seroprevalence has a significant impact on the paediatric epidemiology of these infections, while children frequently transmit herpesviruses to their mothers.

Little is known about differences in prevalence by ethnic group. CMV seroprevalence is generally around 40-50% among white populations but as high as 80-100% in some non-white populations [13]. In a study conducted in London, UK 20 years ago, 46% of white women were CMV seropositive compared to 88% of Asian women and 77% of black women [14]. Similar ethnic variations in CMV seroprevalence have been observed in the Netherlands [15] and the US [16]. There were several other UK studies in the 1980s and 1990s to estimate seroprevalence but no recent studies and it is not known whether seroprevalence has changed. 

EBV seroprevalence among adults is high at around 80-90% in most populations [17,18]. In contrast to CMV and EBV, VZV infection is acquired at older ages in many tropical countries compared to the UK. Seroprevalence among pregnant women is therefore often lower for those born in Asia. In a study in East London between 2001 and 2004, VZV seroprevalence was 93.1% in white British women, 95.2% in UK-born Bangladeshi women and only 84.6% in women born in Bangladesh [19].

The aim of this study was to estimate the seroprevalence of CMV, EBV and VZV among pregnant women in Bradford by ethnic group and country of birth.

## Methods

Born in Bradford (BiB) is a longitudinal multi-ethnic birth cohort study aiming to examine the impact of environmental, psychological and genetic factors on maternal and child health and wellbeing [20]. Bradford is a city in the North of England with high levels of socio-economic deprivation and ethnic diversity. Approximately half of the births in the city are to mothers with South Asian origin and around half of these women were born outside the UK and migrated for marriage.

Women were recruited while waiting for their glucose tolerance test, a routine procedure offered to all pregnant women registered at the Bradford Royal Infirmary, at 26-28 weeks gestation. For those consenting, a baseline questionnaire was completed via an interview with a study administrator.

The baseline questionnaire for the mothers was transliterated into Urdu and Mirpuri using a standardized process, so that words and phrases corresponded with the original English version [21]. As Mirpuri does not have a written form trained bilingual interviewers administered the transliterated questionnaires to Mirpuri speakers. The questionnaire asked about current housing, household composition, marital status, education and current employment of both the mother and the baby’s father, household income, smoking, alcohol and drug use, diet, exercise and general health. 

Each woman was asked to which ethnic group she considered she belonged from the list: White, Asian or Asian British, Mixed ethnic group, Chinese, Black or Black British, or Other. She was also asked to report her cultural background; for example, for those reporting White as their ethnic group the options were British, Irish or Other and for those reporting Asian or Asian British as their ethnic group the cultural background options were Indian, Pakistani, Bangladeshi, Indian Caribbean, African Indian or Other. There were detailed questions about the mother’s and baby’s father’s country of birth and that of their parents and grandparents.

The full BiB cohort recruited 12,453 women during 13,776 pregnancies between 2007 and 2010, 85% of those eligible [22], and the cohort is broadly characteristic of the city’s maternal population. 

The Allergy and Infection Study (ALL IN) is a sub-study of BiB which aims to describe the maternal and paediatric epidemiology of CMV, EBV and VZV and to investigate the effect of age at infection with these viruses on immune function and atopic allergy. Babies born since March 2008 and their mothers who were already enrolled in BiB have been recruited to ALL IN. A venous blood sample was collected from each mother at recruitment to BiB at 24-28 weeks’ gestation and a serum aliquot was stored at -80°C.

A stratified random sample of all BiB mothers with a baseline questionnaire and a serum sample available since August 2008 (when separate aliquots were stored for ALL IN), n=3837, was selected to include 350 white, UK born women, 300 Asian/Asian British, UK born women and 300 Asian/Asian British women born in South Asia who moved to the UK at 10 years of age or older. These sample sizes were estimated to provide power to determine biologically important differences between groups. 

The serum samples for these women were analysed at the Royal Free Virology laboratory with chemiluminescence assays for CMV IgG (Abbott), EBV IgG (Diasorin) and VZV IgG (Diasorin). Briefly, the Abbott Architect i2000sr analyser used CMV IgG kit product no 6C15-20 and protocol 531, according to manufacturer’s instructions. The first step in this automated immunoassay was addition of 25 μl of sample, assay diluent, and paramagnetic microparticles coated with CMV lysate (strain AD169) to a reaction vessel. Controls and calibrators were run in advance of the testing, calibrations were performed every 30 days and controls were run daily. Indirect chemiluminscence immunoassays for VZV IgG and EBV VCA IgG antibodies were performed using the DiaSorin Liaison Analyser using EBV VCA-G kit ID 310510 and VZVG kit ID 058013. Kit controls were run daily and the calibrations were run according to manufacturers’ instructions. 20μl sample was required for each test. For EBV, the magnetic particles were coated in p18 synthetic peptide and a mouse monoclonal antibody linked to an isoluminol-antibody conjugate was used; for VZV, varicella-zoster virus antigen coated the magnetic particles and a mouse monoclonal antibody to human IgG was linked to isoluminol-antibody conjugate. 

### Ethics statement

Ethical approval for this study was obtained from the Bradford Research Ethics Committee (ref: 08/H1302/21) and the London School of Hygiene and Tropical Medicine Ethics Committee (ref: 2008/5320). Women gave written informed consent before taking part.

### Statistical methods

Data were analysed using Stata version 12.1 (StataCorp. 2011. *Stata Statistical Software: Release 12*. College Station, TX: StataCorp LP).

The analysis accounted for the stratified sampling design by using svy commands in Stata. Associations between socio-demographic variables and ethnic group/country of birth categories or seroprevalence were tested with the design-based F statistic. The variables included in the analysis were identified from the literature as potential risk factors for seroprevalence.

Adjusted risk ratio estimates were obtained using Poisson regression adjusted by chi-squared. This method has been shown to be a better alternative to logistic regression to directly estimate the prevalence ratio [23]. 

## Results

### Socio-demographic characteristics by ethnic group/country of birth category

One mother withdrew from BiB after the random sampling was done so blood samples from 949 women were analysed. Among the 350 white UK born women, 346 were British and 4 were Irish, hereafter referred to as White British. Of the 300 Asian UK born women, 267 considered their cultural background as Pakistani, 25 Indian, 6 Bangladeshi, one as British and one as Kenyan Asian, hereafter referred to as South Asian, UK born. Among the 299 Asian women born in South Asia, 270 considered their cultural background to be Pakistani, 25 Indian and 4 Bangladeshi, hereafter referred to as South Asian, South Asia born [24]. Table 1 shows the socio-demographic characteristics of the women in each of the ethnic group/country of birth categories. The mean age at recruitment of the women in each group was 26.6 years (SD 5.7), 27.4 years (SD 4.9) and 28.1 years (SD 5.2) for White British, South Asian UK born and South Asian South Asia born, respectively. There was a pattern of increasing parity, maternal age and household size across the groups, lowest among the white women and highest among the women born in South Asia. South Asian women were significantly more likely to be married than the white women. Among the women born in South Asia, the median age at moving to the UK was 21 years (range 10 - 37). Only 17/299 (6%) moved before age 17.

**Table 1:** Socio-demographic variables by ethnic group/country of birth category.

		**White British n=350**	**South Asian UK born n=300**	**South Asian S. Asia born n=299**	**Design-based F, p**
**Parity**	0	158 (45%)	109 (36%)	85 (28%)	10.54, <0.0001
	1	115 (33)	89 (30)	80 (27)	
	2	48 (14)	54 (18)	68 (23)	
	3+	29 (8)	48 (16)	66 (22)	
**Maternal**	<20	41 (12)	14 (5)	9 (3)	6.51, <0.0001
**age**	20-24	102 (29)	69 (23)	74 (25)	
	25-29	93 (26)	124 (41)	107 (36)	
	30-34	77 (22)	68 (23)	72 (24)	
	35+	37 (11)	25 (8)	37 (12)	
**Household**	1-2	136 (39)	45 (15)	25 (8)	39.08, <0.0001
**size**	3	115 (33)	53 (18)	45 (15)	
	4	55 (16)	59 (20)	59 (20)	
	5-7	42 (12)	110 (36)	123 (41)	
	8+	2 (1)	33 (11)	47 (16)	
**Marital**	Married	123 (35)	284 (95)	295 (99)	
**status**	Single	214 (61)	10 (3)	0	161.44, <0.0001
	Separated/divorced	13 (4)	5 (2)	4 (1)	
	missing	0	1	0	
**Ever**	Yes	97 (76)	111 (65)	32 (12)	130.14, <0.0001
**worked**	No	31	60	240	
	missing	222	129	27	

The socio-demographic characteristics of the women in this stratified random sample were compared with the BiB cohort overall [20] and were broadly similar with respect to maternal age, marital status and household size (data not shown).

### Seroprevalence of CMV, EBV and VZV

732 women were seropositive for CMV and 217 women were seronegative. 888 women were EBV seropositive and 61 were negative. 878 women were seropositive for VZV, 64 were negative and the result was equivocal for 7 women. The seroprevalence estimates in Table 2 account for the stratified sampling design. Less than half of the White British women were CMV seropositive while seroprevalence was high among the South Asian women, even those born in the UK. VZV seroprevalence was high among the women born in the UK regardless of ethnic group and significantly lower among the women born in South Asia. EBV seroprevalence was 93.6% overall and did not vary by ethnic group/country of birth category.

**Table 2:** Seroprevalence of CMV, EBV and VZV by ethnic group/country of birth category and other socio-demographic variables.

		**Seroprevalence (%)**					
		**CMV**	p*	**EBV**	**p**	**VZV**	**p**
**n**		949		949		942	
	All	72.9		93.6		93.4	
**Ethnic**	White British	48.6	<0.0001	93.7	0.64	94.8	0.003
**group/CoB**	South Asian, UK born	89.3		94.3		95.0	
**category**	South Asian, S. Asia born	98.3		92.6		89.6	
**Parity**	0	62.5	<0.0001	93.3	0.99	90.6	0.002
	1	74.8		93.9		93.9	
	2	82.5		93.3		94.1	
	3+	85.3		93.9		98.7	
**Maternal**	<20	50.9	0.0002	91.6	0.61	86.2	0.01
**age**	20-24	73.4		92.3		92.5	
	25-29	76.3		93.6		93.2	
	30-34	73.5		95.0		94.9	
	35+	75.5		94.8		97.9	
**Household**	1-2	53.2	<0.0001	92.0	0.50	95.2	0.09
**size**	3	65.9		95.0		95.1	
	4	78.9		92.3		92.3	
	5-7	87.1		94.4		92.4	
	8+	94.7		94.6		88.1	
**Marital**	Married	82.5	<0.0001	94.6	0.14	92.9	0.31
**status**	Single	50.9		91.5		94.1	
(1 missing)	Separated/divorced	68.5		89.5		100.0	

* p values are for design-based F statistic (test of association between socio-demographic variables and seroprevalence allowing for stratified sampling design)

For CMV there were differences in seroprevalence between cultural background groups for South Asian women. Among the UK born, 68% of Indian women were seropositive compared to 91% of Pakistani women (design-based F=19.03, p<0.0001). For those born in South Asia, 92% of Indian women were seropositive compared to 99% of Pakistani women (design-based F=9.02, p=0.003).

There was a trend of increasing CMV seroprevalence with increasing parity, age and household size (Table 2). VZV seroprevalence also rose with increasing parity and maternal age while there was no effect of any of these variables on EBV prevalence. 

Due to differences in socio-demographic factors by ethnic group/country of birth category, the patterns of seroprevalence by parity, maternal age and household size were examined separately for each group. While there was a strong trend of increasing CMV seroprevalence with increasing parity in White British women (39% among nulliparous women to 62% in those with 3 or more children), there was less variation by parity among the South Asian, UK born women; 85% of nulliparous women were seropositive and the highest seroprevalence was 93% among women with one previous child. The women born in South Asia had high CMV seroprevalence (>95%) regardless of parity.

There was no clear trend of increasing CMV seroprevalence with increasing maternal age in White British women and seroprevalence was high for South Asian women of all ages. The oldest group of women born in South Asia (35+ years) had significantly lower seroprevalence (92% vs. 99% or 100% in the other age groups, p=0.004). Household size was significantly associated with CMV seroprevalence only among South Asian, UK born women (78% in smallest households to 94% in larger households, p=0.0009) but no clear trend was observed.

Conversely, the trend of increasing VZV seroprevalence with increasing parity and maternal age was only seen among the women born in South Asia; among the women born in the UK VZV seroprevalence was high in all groups. Household size was only associated with VZV seroprevalence in the UK-born South Asian women where it was significantly lower in women living in households of 8 or more people (85% vs. 95% or 98% in the other groups, p=0.01).

### Ethnic group/country of birth category and CMV

The association between ethnic group/country of birth category and CMV seropositivity remained after adjustment for parity and household size (Table 3). South Asian women born in the UK were 1.8 times more likely to be CMV seropositive than White British women and South Asian women born in South Asia were nearly twice as likely to be CMV seropositive than White British women.

**Table 3:** Association of ethnic group/country of birth category and CMV seropositivity (n=949).

		**Unadjusted risk ratio (95% CI, p)**	**Adjusted risk ratio (95% CI, p)**
**Ethnic**	White British	1.0	1.0
**group/CoB**	South Asian, UK born	1.84 (1.68 to 2.02), <0.001	1.76 (1.60 to 1.94), <0.001
**category**	South Asian, S. Asia born	2.02 (1.85 to 2.82), <0.001	1.91 (1.73 to 2.11), <0.001
**Parity**	0	1.0	1.0
	1	1.17 (1.07 to 1.27), 0.001	1.13 (1.02 to 1.25), 0.02
	2	1.26 (1.14 to 1.39), <0.001	1.12 (0.98 to 1.26), 0.09
	3+	1.29 (1.17 to 1.43), <0.001	1.06 (0.95 to 1.19), 0.30
**Maternal age**	<20	1.0	-
	20-24	1.37 (1.16 to 1.63), <0.001	
	25-29	1.43 (1.21 to 1.69), <0.001	
	30-34	1.38 (1.17 to 1.65), <0.001	
	35+	1.40 (1.16 to 1.69), <0.001	
**Household size**	1-2	1.0	1.0
	3	1.24 (1.10 to 1.39), <0.001	1.05 (0.92 to 1.21), 0.44
	4	1.44 (1.28 to 1.61), <0.001	1.09 (0.94 to 1.26), 0.28
	5-7	1.58 (1.42 to 1.75), <0.001	1.13 (1.00 to 1.28), 0.05
	8+	1.67 (1.46 to 1.92), <0.001	1.12 (0.96 to 1.30), 0.15
**Marital status**	Married	1.0	-
(1 missing)	Single	0.60 (0.55 to 0.66), <0.001	
	Separated/divorced	0.85 (0.67 to 1.08), 0.18	

Including all variables in Table 3 in the model did not alter the results substantially; adjusted risk ratios for South Asian, UK born and South Asian, South Asia born were 1.84 (95% CI 1.62 to 2.09, p<0.001) and 2.00 (95% CI 1.75 to 2.28, p<0.001) respectively (n=948).

Given the differences in CMV seroprevalence between Pakistani and Indian women, the multivariable analysis in Table 3 was re-run excluding the small groups of non-Pakistani South Asian women, i.e. to compare White British women with UK born Pakistani women and South Asian born Pakistani women (n=887). There were no substantial differences in the unadjusted or adjusted risk ratios.

To investigate the high CMV seroprevalence among South Asian women born in the UK we analysed the data on country of birth of their mothers. 254 (85%) were born in Pakistan, 21 (7%) in India, 6 (2%) in Bangladesh, 9 (3%) elsewhere (Kenya 7, Uganda 1, Burma 1) and only 10 (3%) in England or Wales. 

### Ethnic group/country of birth category and VZV

VZV seroprevalence was significantly lower among the South Asian women born in South Asia than White British women after adjusting for maternal age (Table 4). Also including parity in the model did not change the estimates substantially; adjusted risk ratios for South Asian, UK born and South Asian, South Asia born were 0.99 (95% CI 0.95 to 1.03, p=0.66) and 0.93 (95% CI 0.89 to 0.97, p=0.001) respectively (n=942).

**Table 4:** Association of ethnic group/country of birth category and VZV seropositivity (n=942).

		**Unadjusted risk ratio (95% CI, p)**	**Adjusted risk ratio (95% CI, p)**
**Ethnic**	White British	1.0	1.0
**group/CoB**	South Asian, UK born	1.00 (0.96 to 1.04), 0.95	1.00 (0.95 to 1.04), 0.85
**category**	South Asian, S. Asia born	0.94 (0.91 to 0.99), 0.008	0.94 (0.90 to 0.98), 0.002
**Parity**	0	1.0	-
	1	1.04 (0.99 to 1.08), 0.09	
	2	1.04 (0.99 to 1.09), 0.12	
	3+	1.09 (1.04 to 1.15), 0.001	
**Maternal age**	<20	1.0	1.0
	20-24	1.07 (0.99 to 1.16), 0.07	1.08 (1.00 to 1.17), 0.04
	25-29	1.08 (1.00 to 1.16), 0.05	1.09 (1.01 to 1.18), 0.02
	30-34	1.10 (1.02 to 1.19), 0.01	1.12 (1.03 to 1.21), 0.005
	35+	1.14 (1.05 to 1.24), 0.003	1.16 (1.06 to 1.26), 0.001
**Household size**	1-2	1.0	-
	3	1.00 (0.95 to 1.05), 0.96	
	4	0.97 (0.92 to 1.03), 0.29	
	5-7	0.97 (0.92 to 1.02), 0.23	
	8+	0.92 (0.86 to 0.99), 0.03	
**Marital status**	Married	1.0	-
(1 missing)	Single	1.02 (0.98 to 1.06), 0.45	
	Separated/divorced	1.08 (0.96 to 1.21), 0.20	

## Discussion

Our results demonstrate differences in the seroprevalence of CMV and VZV among pregnant women in Bradford according to ethnic group and country of birth. These differences remained after adjustment for socio-demographic differences between the groups. 

The large BiB cohort provided a unique opportunity to investigate variation in seroprevalence by ethnic group and country of birth and the stratified design ensured sufficient statistical power to compare the three ethnic group/country of birth categories. Detailed socio-demographic data were available allowing the effect of these factors to be investigated and adjusted for. Seroprevalence of CMV has been associated with socio-economic deprivation in some studies [13] but not in others [25]. As Bradford has higher levels of deprivation than the UK national average [20], it is possible that our estimates of seroprevalence are higher than might be found elsewhere in the UK. However, we are confident of the differences we have observed by ethnic group and country of birth because we have adjusted for several socio-demographic factors which are likely to be more proximal risk factors for seroprevalence than general measures of socio-economic status such as household income. 

The CMV seroprevalences we observed among White British and South Asian women were similar to those reported by Tookey et al. [14], 46% in white women and 88% in Asian women. They did not report seroprevalence in Asian women separately by country of birth but stated that ‘British born women of all ethnic groups were less likely to be seropositive than those born elsewhere’ [14]. Their multivariable analysis was restricted to white women born in the British Isles to investigate the association of parity and seroprevalence so they did not adjust for socio-demographic variables when comparing ethnic groups. Staras et al. [16] found ethnic differences in CMV seroprevalence, 51% among non-Hispanic white people compared to 76% and 82% among the non-Hispanic black and Mexican American groups, respectively. These differences persisted after allowing for socio-demographic factors including country of birth and were observed in the sub-group of women of childbearing age. They did not specifically compare seroprevalence in black and Mexican Americans born in and outside the US but found that overall those born in other countries were more likely to be seropositive than those born in the US.

Our VZV seroprevalence data were similar to those of Talukder et al. who found that 93% of white British women were VZV seropositive as were 95% of UK-born Bangladeshi women and 85% of Bangladesh-born women [19]. Bangladesh-born women who migrated to the UK after the age of 15 years were significantly less likely to be VZV seropositive compared to the white UK-born women. However, seroprevalence among Bangladeshi women arriving in the UK aged 15 years or less or those born in the UK was not significantly lower than that of the white UK-born group. In our study we restricted the group of South Asian women born in South Asia to those who moved to the UK aged 10 years or older to distinguish between childhood and adulthood exposure. 

Our results show that ethnic group and country of birth have a greater influence on CMV serostatus than factors such as parity and household size, suggesting that early life exposures, in particular breastfeeding, are more important than current adulthood exposures. This is supported by the high seroprevalence among Asian women born in the UK and that 97% of their mothers were born outside the UK. In the BiB cohort Asian women are more likely to breastfeed and for longer than white British women [26]; it is likely that this difference also existed in the previous generation and explains the higher CMV prevalence in Asian women. The high CMV seroprevalence among UK-born South Asian women is likely to fall only slowly over subsequent generations as CMV seroprevalence in the children of these women is expected to be higher than that of the White British children. We expect the South Asian children to be infected with CMV earlier than the white children as the former are likely to acquire infection from their mothers through breastfeeding and close contact whereas children born to seronegative mothers will become infected through contact with other children. 

We found a trend of increasing CMV prevalence with increasing parity among the white women suggesting that child to mother transmission is important in this group, a similar finding to Tookey et al. [14]. The lack of a significant association with parity among the Asian women is likely to be because many had already acquired the infection in childhood from their mothers and infection from their own children was less common. In contrast to CMV, VZV seroprevalence among South Asian women born in the UK was the same as for the White British women. This reflects that most VZV infections are acquired from other children at nursery or school rather than through close maternal contact.

It is possible that in multi-ethnic populations such as Bradford, the high CMV seroprevalence in the South Asian community could increase seroprevalence in the White British population, at least among the children as there is likely to be greater contact between children at nursery and school than between mothers. Increased child to mother transmission may then result in a greater chance of primary infection in pregnancy in white seronegative women. In the Netherlands CMV seroprevalence was higher in native Dutch women living in Amsterdam and Rotterdam, 50% and 55% respectively, than in the south-east where it was 35%. Nearly half of the women living in Amsterdam and Rotterdam are non-native compared to 11% in south-eastern Netherlands [15]. This may reflect greater transmission of the virus in the multi-ethnic communities but may also be associated with different socio-demographic characteristics of the metropolitan region compared to the south-east.

The South Asian women are clearly genetically susceptible to VZV infection, because they readily acquire this infection in the UK. The finding of lower seroprevalence in South Asian women born in Asia is thus compatible with less efficient transmission of chickenpox in the Asian environment with humidity, high temperature and ultraviolet light potentially reducing the infectiousness of chickenpox [27,28]. South Asian women born in South Asia are at risk of VZV infection during pregnancy which could produce congenital varicella syndrome or perinatal chickenpox. 

About half of the White British women in Bradford are at risk of primary CMV infection in pregnancy and the associated increased risk of disease due to congenital infection. Children born to South Asian women are more likely to acquire congenital CMV infection from mothers who have natural immunity before they become pregnant. Although this maternal immunity may modulate the severity of fetal disease caused by CMV, the high abundance of seropositive women means that more babies with congenital CMV infection are born to immune women than to those who enter pregnancy susceptible to primary infection [29]. In the USA, approximately 75% of babies with congenital CMV infection are born to immune women [3]. An analysis of data from the UK and Sweden showed that around half of children with moderate or severe outcomes of congenital CMV infection had mothers with confirmed or presumed non-primary infection [30]. In addition to its effects in pregnancy, recent evidence suggests an association between CMV seropositivity and increased mortality [31] and CMV may play a role in immunosenescence [32]. 

This study confirms differences in CMV and VZV seroprevalence by ethnic group at a time when vaccination programmes for these viruses are being considered. In addition, we demonstrate the importance of country of birth in determining serostatus. It has been proposed that a vaccination programme for CMV would include vaccination of toddlers (to reduce transmission to their mothers) and of male and female 12 year-olds [10]. In predicting the impact of such a vaccination programme it is important to recognise that a large proportion of children born to South Asian women may have already acquired CMV from their mothers at the time of scheduled vaccination. Prototype CMV vaccines have been shown to boost natural immunity and this effect could be important if it provides protection against reinfection and/or reduces the contagiousness of individuals in the community [33-35].
